# Modulation indices and plan delivery accuracy of volumetric modulated arc therapy

**DOI:** 10.1002/acm2.12589

**Published:** 2019-04-30

**Authors:** Jong Min Park, Jung‐in Kim, So‐Yeon Park

**Affiliations:** ^1^ Department of Radiation Oncology Seoul National University Hospital Seoul Korea; ^2^ Institute of Radiation Medicine Seoul National University Medical Research Center Seoul Korea; ^3^ Biomedical Research Institute Seoul National University Hospital Seoul Korea; ^4^ Institute for Smart System Robotics Research Laboratory for Extreme Environments Advanced Institutes of Convergence Technology Suwon Korea; ^5^ Department of Radiation Oncology Veterans Health Service Medical Center Seoul Korea

**Keywords:** gamma passing rate, modulation index, plan delivery accuracy, volumetric modulated arc therapy

## Abstract

**Purpose:**

We evaluated the performance of various modulation indices (MI) for volumetric modulated arc therapy (VMAT) to predict plan delivery accuracy.

**Methods:**

The specific indices evaluated were MI quantifying the mechanical uncertainty (MI_t_), MI quantifying the mechanical and dose calculation uncertainties (MI_c_), MI for station parameter optimized radiation therapy (MI_SPORT_), modulation complexity score for VMAT (MCS_v_), leaf travel modulation complexity score (LTMCS), plan averaged beam area (PA), plan averaged beam irregularity (PI), plan averaged beam modulation (PM), and plan normalized monitor unit (PMU) to predict VMAT delivery accuracy. By utilizing 240 VMAT plans generated with the Trilogy and TrueBeam STx, Spearman's rank correlation coefficients (*r*) were calculated between the MIs and measures of conventional methods.

**Results:**

For the Trilogy system, MI_c_ showed the highest *r* values with gamma passing rates (GPRs) (*r* = −0.624 with *P *< 0.001 for MapCHECK2 and *r* = −0.655 with *P *< 0.001 for ArcCHECK). For TrueBeam STx, MI_c_ also showed the highest *r* values with GPRs (*r* = −0.625 with *P *< 0.001 for the MapCHECK2 and *r* = −0.561 with *P *< 0.001 for the ArcCHECK). The MI_t_ and MI_c_ showed the highest *r* values to the MLC position errors for the Trilogy and TrueBeam STx systems (*r* = 0.770 with *P *< 0.001 and *r* = 0.712 with *P *< 0.001, respectively). The PA showed the highest percent of *r* values (*P* < 0.05) to differences in the dose‐volume parameters between original VMAT plans and actual deliveries for the Trilogy systems (30.9%). Both the MI_t_ and MI_c_ showed the highest percent of *r* values (*P* < 0.05) to differences in the dose‐volume parameters between original VMAT plans and actual deliveries for the TrueBeam STx systems (31.8%).

**Conclusion:**

To comprehensively review the results, the MI_c_ showed the best performance to predict the VMAT delivery accuracy.

## 1 INTRODUCTION

Advanced radiotherapy techniques, such as intensity‐modulated radiation therapy (IMRT) and volumetric modulated arc therapy (VMAT), facilitate conformal deliveries of prescription doses to target volumes, while minimizing doses to normal tissue proximal to the target volumes with intensity modulation.^1^, ^2^ Moreover, the intensity modulation of IMRT and VMAT enables the generation of steep dose gradients between the target volumes and particular organs at risk (OARs) close to the target volumes.^3^ This could reduce radiotherapy‐induced complications, as well as escalate the prescription doses to increase the therapeutic effect of radiotherapy.^3^ Especially, VMAT can rapidly deliver equal or superior dose distributions, compared to those of IMRT by simultaneous modulations of multi‐leaf collimator (MLC) movements, gantry speeds, and dose‐rates.^1^, ^3^ However, the intensity modulation increases the uncertainty of the planned dose delivery to a patient during actual treatment, which is an adverse effect of IMRT or VMAT.^4^ Since high intensity modulation is involved in the complicated mechanical movements of the linac, such as MLC movements, and the frequent use of small or irregular fields with relatively low dose calculation accuracy, there might be a clinically significant discrepancy between the intended treatment plan and its actual delivery in highly modulated IMRT or VMAT plans.^5^, ^6^, ^7^, ^8^ In this respect, patient‐specific pre‐treatment quality assurance (QA) according to international guidelines is highly recommended to verify plan delivery accuracy before patient treatment for both IMRT and VMAT.^4^, ^9^, ^10^, ^11^

As a patient‐specific pre‐treatment QA, 2D gamma analysis between the measured planar dose distributions with 2D dosimeters and the calculated dose distributions in the treatment planning system (TPS) is widely adopted in clinical settings.^12^, ^13^ Although gamma analysis is a practical and convenient method to evaluate the similarity of two distributions, recent studies pointed out the clinical irrelevance of the gamma passing rates.^14^, ^15^ As an alternative method for patient‐specific pre‐treatment QA according to gamma analysis, several studies recommended that the recorded log files in the linac control system during beam delivery be analyzed.^11^, ^14^, ^16^, ^17^, ^18^ However, this method has an intrinsic disadvantage in that it is dependent on the linac control system. In addition, it is hard to determine the clinically relevant tolerance levels for each VMAT mechanical parameter for the linac log file analysis method. On the other hand, several studies suggested a modulation index as a patient‐specific pre‐treatment QA method by quantification of the modulation degree of VMAT plans.^5^, ^6^, ^8^, ^19^, ^20^ The modulation index is advantageous in terms of efficiency since it can be calculated at the planning level, which reduces resources in the clinic. Li and Xing suggested a modulation index (MI_SPORT_) by quantifying movements of MLCs weighted by segmental monitor unit (MU) for VMAT.^5^ They did not demonstrate the performance of MI_SPORT_ as a pre‐treatment QA method for VMAT but only used MI_SPORT_ as a tool to suggest station parameter optimized radiation therapy (SPORT). Masi et al. suggested the modulation complexity score for VMAT (MCS_v_) and leaf travel modulation complexity score (LTMCS).^6^ These indicators were modifications of the modulation complexity score (MCS), which was originally suggested by McNiven et al. to evaluate the modulation degree of IMRT plans.^6^ Du et al. suggested several modulation indices for VMAT, which were plan averaged beam area (PA, average area of beam apertures), plan averaged beam irregularity (PI, deviations of the aperture shapes from a circle), plan averaged beam modulation (PM, extent of a large open field being broken into multiple small segments), and plan normalized monitor units (PMU, MU normalized by the fractional prescription dose).^19^ The modulation indices by Du et al. focused on the calculation of dose uncertainties due to frequent use of irregular or small beam segments rather than using mechanical uncertainties during plan delivery. We also suggested a modulation index, which evaluates the modulation of VMAT mechanical parameters (MI_t_) by analyzing the speed and acceleration of MLC movements, gantry rotation variations, and dose‐rate variations.^8^ Furthermore, we suggested a modulation index that considers both the mechanical parameter modulations and irregularity of the beam aperture shapes defined by the MLCs with the thinning algorithm (MI_c_).^20^ Although various modulation indices for VMAT were suggested in the previous studies, a comprehensive performance test on the previously suggested modulation indices for VMAT has not yet been performed. Therefore, in this study, we tested the performance of various modulation indices by utilizing VMAT plans with various tumor sites. Correlations between values of each modulation index and (1) the gamma passing rates, (2) differences in the mechanical parameters between VMAT plans and delivery records, and (3) differences in the clinically relevant dose‐volumetric parameters between the original VMAT plans and the plans reconstructed from the delivery records were analyzed to evaluate the performance of the various modulation indices. We utilized two types of linacs and two types of dosimeters in this study.

## 2 MATERIALS AND METHODS

### 2.1 Patient selection

For this study, a total of 200 patients with head and neck (H&N) cancer, prostate cancer, liver cancer, lung cancer, brain tumor, and spine tumor were retrospectively selected after an institutional review board approval (IRB No. 1802‐069‐922). Every patient underwent CT scans using the Brilliance CT Big Bore™ system (Phillips, Amsterdam, Netherlands).

### 2.2 VMAT planning

In this study, a total of 140 and 100 VMAT plans with two arcs were generated for the Trilogy™ system with the Millennium 120™ MLC and the TrueBeam STx™ system with the high‐definition (HD) 120™ MLC (Varian Medical Systems, Palo Alto, CA, USA), respectively.

Forty plans for H&N cancer, 40 primary plans for prostate cancer, 40 boost plans for prostate cancer, 11 plans for liver cancer, and nine plans for spine tumors were generated with the Trilogy system, that is, C‐series linac. For H&N VMAT plans, 6 MV photon beams were used while 15 MV photon beams were used for the other VMAT plans. The simultaneous integrated boost (SIB) technique with a total of three planning target volumes (PTVs) was used for H&N VMAT plans with prescription doses of 67.5 Gy (daily dose = 2.25 Gy) to the PTV_67.5_, 54 Gy (daily dose = 1.8 Gy) to the PTV_54_, and 48 Gy (daily dose = 1.6 Gy) to the PTV_48_ (30 fractions). For prostate cancer, a primary plan for each patient were generated with a prescription dose of 50.4 Gy (daily dose = 1.8 Gy, 28 fractions) and a boost plan with a prescription dose of 30.6 Gy (daily dose = 1.8 Gy, 17 fractions). For liver cancer, the prescription dose was 50 Gy in 25 fractions (daily dose = 2 Gy). For spine tumor, the prescription dose was 30 Gy in 10 fractions (daily dose = 3 Gy).

Twenty VMAT plans for H&N cancer, 20 VMAT plans for brain tumor, 20 stereotactic ablative radiotherapy (SABR) VMAT plans for lung cancer, 20 VMAT plans for spine SABR, and 20 VMAT plans for liver SABR were generated with the TrueBeam STx system. Just as in the H&N VMAT plans with the C‐series linac, the SIB technique was used for H&N VMAT plans with the TrueBeam STx system with the same target volumes and prescription doses using six MV photon beams. For brain VMAT plans, six MV photon beams were used to deliver the prescription dose of 30 Gy in ten equal fractions (daily dose = 3 Gy). For lung SABR, flattening filter free six MV (6 FFF) photon beams were used to deliver a prescription dose of 60 Gy in four equal fractions (daily dose = 15 Gy). For both spine SABR and liver SABR, ten FFF photon beams were used to deliver 16 Gy in entirety and 42 Gy in three equal fractions (daily dose = 14 Gy), respectively.

For the Trilogy system, all VMAT plans were generated using two full arcs. For the TrueBeam STx system, H&N VMAT and spine SABR plans used two full arcs while brain VMAT, liver SABR, and lung SABR plans used two partial arcs, depending on the target position, target size, and positional relationship between the target volume and OARs.

For every VMAT plan in this study, the progressive resolution optimizer (PRO, Varian Medical Systems, Palo Alto, CA, USA) in the Eclipse™ system (ver.13.7, Varian Medical Systems, Palo Alto, CA, USA) was used to optimize VMAT plans. For dose calculation, the anisotropic analytic algorithm (AAA, Varian Medical Systems, Palo Alto, CA, USA) in the Eclipse system was used with a dose calculation grid size of 1 mm.

### 2.3 Gamma evaluation

For each VMAT plan, verification plans were generated to determine reference dose distributions for gamma evaluation with the MapCHECK2™ dosimeter inserted in the MapPHAN™ (Sun Nuclear Corporation, Melbourne, FL, USA) and the ArcCHECK™ (Sun Nuclear Corporation, Melbourne, FL, USA). The reference dose distributions were calculated with a dose calculation grid size of 1 mm. For each VMAT plan, local gamma evaluations with absolute doses were performed using both the MapCHECK2 and the ArcCHECK arrays. Before VMAT dose distribution measurements, the output of the linacs were calibrated according to the American Association of Physicists in Medicine (AAPM) Task Group (TG) 51 protocol.^21^ Both the MapCHECK2 and the ArcCHECK arrays were calibrated according to the manufacturer guidelines before performing VMAT dose distribution measurements. For the local gamma evaluation, gamma criteria of 3%/3 mm, 2%/2 mm, 2%/1 mm, 1%/2 mm, and 1%/1 mm were used. Doses equal to or <10% of the prescription dose was ignored when calculating gamma passing rates.^4^, ^10^ SNC software (Sun Nuclear Corporation, Melbourne, FL, USA) was used to calculate local gamma passing rates for both the MapCHECK2 and ArcCHECK measurements.

### 2.4 Log file analysis

When delivering VMAT plans for dose distribution measurements using the MapCHECK2 and the ArcCHECK for gamma evaluation, the actual MLC positions, gantry angles, and the delivered MUs at each control point during beam delivery were acquired using the log files recorded in the linac control system. The log files were reformatted as DICOM‐RT files with an in‐house program written in MATLAB (Mathworks Inc., Natick, MA, USA). The DICOM‐RT formatted log files were compared to the original VMAT plans generated in the Eclipse system, and the differences in the MLC positions, gantry angles, and delivered MUs between the original VMAT plans and the log files were calculated for each VMAT plan. Since the VMAT delivery occurred once for each 2D dosimeter (MapCHECK2 and ArcCHECK arrays), two DICOM‐RT formatted log files were acquired for a single VMAT plan. Therefore, we acquired two sets of differences in the mechanical parameters and averaged the differences for each VMAT plan.

### 2.5 Dose‐volumetric parameter differences between the original VMAT plans and the VMAT plans reconstructed with log files

The DICOM‐RT formatted log files were imported into the Eclipse system, and dose distributions were calculated in the patient CT images used for generating the original VMAT plan. When calculating dose distributions from the log files, the dose calculation grid size was kept identical to that of original VMAT plan calculation (1 mm). Clinically relevant dose‐volumetric parameters under previous studies and guidelines were calculated with the original VMAT plan, as well as VMAT plans reconstructed from the log files.^22^, ^23^ The differences in the dose‐volumetric parameters between the dose distributions reconstructed with the log files and those of the original VMAT plans were acquired. Since there were two sets of log files (MapCHECK and ArcCHECK2 measurements) for each VMAT plan, two sets of differences in the dose‐volumetric parameters were acquired. We averaged those differences for each VMAT plan. For H&N VMAT plans, a total of 48 dose‐volumetric parameters were examined (Table S1). For prostate VMAT plans, a total of 29 dose‐volumetric parameters were examined for both primary and boost plans (Table S1). For brain, liver, and spine VMAT plans (not SABR), 27, 22, and 24 dose‐volumetric parameters were investigated, respectively (Table S1). For lung, spine, and liver SABR VMAT plans, 32, 17, and 33 dose‐volumetric parameters were examined, respectively (Table S1). A total of 309 dose‐volumetric parameters were examined in this study.

### 2.6 Calculation of modulation indices

In this study, a total of nine modulation indices were calculated, which were MI_t_ (*f* = 0.5), MI_c_ (*f* = 0.5), MCS_v_, LTMCS, MI_SPORT_, PA, PI, PM, and PMU. All the modulation indices were calculated with the control point information from the original VMAT plans. In the cases of MI_t_, MI_c_, MCS_v_, and LTMCS, the values of those modulation indices increase as the number of control points increased because those values were acquired by summation of mechanical and dose calculation uncertainties at each control point without any normalization to the number of control points.^6^, ^8^, ^20^ When comparing VMAT plans with the same number of control points, this is not problematic. However, when comparing the VMAT plans with different numbers of control points, this could be problematic because the values of those modulation indices change with the number of control points. For example, low modulation VMAT plans with multiple arcs could show higher values of those modulation indices than did the high modulation VMAT plans with a single arc because of the large number of control points in the VMAT plan with multiple arcs. In this study, the numbers of control points in VMAT plans with various treatment sites were different from one another. Therefore, to eliminate the effect of the control point number on the values of those modulation indices, we divided the values of those modulation indices by the total number of control points for each VMAT plan, that is, the values of those modulation indices were normalized by the number of controls for a fair comparison.

### 2.7 Correlation analysis

To evaluate the performance of the previously suggested modulation indices, Spearman's rank correlation coefficients (*r*) were calculated between the modulation index values and the conventional patient‐specific pre‐treatment QA values, such as gamma passing rates, the differences in the mechanical parameters between calculation and delivery, and dose‐volumetric parameter differences between the original VMAT plans and the VMAT plans reconstructed from the log files. To examine the statistical significance of the values of *r*, we also calculated *P* values for each value of *r*. Correlations of each modulation index were analyzed against the local gamma passing rates with various gamma criteria, the differences in the mechanical parameters (MLC positions, gantry angles, and delivered MUs) between calculation and plan delivery, and the differences in the dose‐volumetric parameters between the original VMAT plans and the VMAT plans reconstructed from the log files. For the dose‐volumetric parameter differences, because a large number of dose‐volumetric parameters were examined in this study (a total of 309 dose‐volumetric parameters), we just calculated the percent of *r* values with corresponding *P* < 0.05, which was regarded as statistically significant in this study.

## 3 RESULTS

### 3.1 Values of the calculated modulation indices

The calculated modulation indices are shown in Table 1.

**Table 1:** Values of modulation indices

Treatment site	N	MI_t_ (×10^−1^)	MI_c_ (×10^−1^)	MCS_v_ (×10^−3^)	LTMCS (×10^−3^)	MI_SPORT_ (×10^6^)	PA (×10^1^)	PI (×10^1^)	PM	PMU (×10^2^)
C‐series linac										
H&N	40	1.35 ± 0.15	1.61 ± 0.19	1.28 ± 0.26	0.52 ± 0.17	5.38 ± 1.53	7.55 ± 2.36	1.67 ± 0.30	0.70 ± 0.06	5.51 ± 1.59
Prostate (PP)	40	0.47 ± 0.07	0.56 ± 0.09	1.47 ± 0.39	0.98 ± 0.32	0.99 ± 0.46	2.01 ± 0.63	0.74 ± 0.16	0.61 ± 0.11	4.94 ± 1.47
Prostate (BP)	40	0.38 ± 0.05	0.45 ± 0.06	1.72 ± 0.39	1.20 ± 0.33	0.60 ± 0.32	1.86 ± 0.54	0.56 ± 0.13	0.49 ± 0.12	4.31 ± 1.20
Liver	11	0.67 ± 0.22	0.79 ± 0.25	1.46 ± 0.26	0.78 ± 0.23	1.88 ± 0.88	4.60 ± 2.87	0.93 ± 0.25	0.62 ± 0.07	4.34 ± 0.63
Spine	9	0.88 ± 0.52	1.05 ± 0.61	1.34 ± 0.38	0.79 ± 0.24	4.88 ± 3.42	4.67 ± 3.61	1.20 ± 0.43	0.69 ± 0.06	6.06 ± 1.37
TrueBeam STx										
Lung (SABR)	20	0.84 ± 0.17	0.98 ± 0.20	3.17 ± 0.36	2.56 ± 0.36	8.50 ± 4.43	1.59 ± 0.74	0.65 ± 0.14	0.53 ± 0.06	5.23 ± 0.57
Spine (SABR)	20	0.56 ± 0.10	0.68 ± 0.12	1.23 ± 0.29	0.78 ± 0.25	3.50 ± 9.24	2.51 ± 1.15	1.25 ± 0.23	0.69 ± 0.06	6.77 ± 1.13
Liver (SABR)	20	0.67 ± 0.35	0.79 ± 0.42	2.15 ± 0.97	1.61 ± 0.83	12.4 ± 7.78	2.76 ± 1.79	0.95 ± 0.34	0.60 ± 0.08	5.52 ± 0.92
Brain	20	1.25 ± 0.54	1.46 ± 0.63	2.45 ± 0.89	1.68 ± 0.85	4.34 ± 1.63	7.48 ± 6.74	0.99 ± 0.27	0.56 ± 0.10	4.05 ± 0.61
H&N	20	1.82 ± 0.18	2.18 ± 0.23	1.16 ± 0.27	0.44 ± 0.16	19.4 ± 3.82	11.7 ±2.44	2.18 ± 0.38	0.74 ± 0.06	6.01 ± 1.70

MI_t_: Modulation index considering mechanical uncertainties in the multi‐leaf collimator (MLC) positions, gantry angle positions, and dose‐rate; MI_c_: Modulation index considering both mechanical uncertainties and dose calculation uncertainty; MCS_v_: Modulation complexity score for volumetric modulated arc therapy (VMAT); LTMCS: Leaf travel modulation complexity score; MI_SPORT_: Modulation index for station parameter optimized radiation therapy; PA: Plan averaged beam area; PI: Plan averaged beam irregularity; PM: Plan averaged beam modulation; PMU: Plan normalized monitor unit; H&N: Head and neck; PP: Primary plan; BP: Boost plan; SABR: Stereotactic ablative radiotherapy.

As VMAT modulation increases, it is known that the values of MI_t_, MI_c_, MI_SPORT_, PI, PM, and PMU increase while the values of MCS_v_, LTMCS, and PA decrease.^5^, ^6^, ^8^, ^20^

For the VMAT plans with the C‐series linac, H&N VMAT plans showed the highest modulation according to every modulation index (except for PA and PMU) since the highest average values of MI_t_, MI_c_, MI_SPORT_, PI, and PM and the lowest average values of MCS_v_ and LTMCS were observed for H&N VMAT plans. Similarly, prostate boost VMAT plans showed the lowest modulation among various types of C‐series linac VMAT plans according to every modulation index in this study, except for the PA. Except PA and PMU, every modulation index showed similar tendency to evaluate the modulation degree for various types of VMAT plans.

For the VMAT plans with the TrueBeam STx system, H&N VMAT plans showed the highest modulation according to every modulation index, except for PA and PMU, since the highest average values of MI_t_, MI_c_, MI_SPORT_, PI, and PM and the lowest average values of MCS_v_ and LTMCS were observed for H&N VMAT plans. In the case of the lowest modulation VMAT plans, MI_t_, MI_c_, and MI_SPORT_ indicated that the modulation degree of the spine SABR VMAT plans were the lowest. However, MCS_v_, LTMCS, PI, and PM indicated that the modulation degree of the lung SABR VMAT plans was the lowest.

### 3.2 Local gamma passing rates

The local gamma passing rates with various gamma criteria of VMAT plans for various treatment sites as measured with the MapCHECK2 and ArcCHECK are shown in Table 2.

**Table 2:** Local gamma passing rates

Treatment site	3%/3 mm		2%/2 mm		2%/1 mm		1%/2 mm		1%/1 mm	
	MC	AC	MC	AC	MC	AC	MC	AC	MC	AC
C‐series linac										
H&N	93.5 ± 1.9	94.9 ± 3.1	85.1 ± 4.2	84.0 ± 5.3	67.3 ± 5.7	63.0 ± 6.4	80.1 ± 5.6	77.2 ± 5.5	58.0 ± 6.4	51.8 ± 5.4
Prostate (PP)	95.8 ± 2.3	96.5 ± 2.6	86.2 ± 5.3	90.6 ± 4.8	63.1 ± 6.4	71.8 ± 9.8	80.0 ± 5.5	86.3 ± 5.2	53.1 ± 6.1	63.4 ± 9.3
Prostate (BP)	96.9 ± 1.7	97.0 ± 2.9	88.7 ± 4.1	91.3 ± 5.9	67.8 ± 5.6	73.6 ± 9.8	82.7 ± 5.1	87.4 ± 6.3	58.2 ± 5.6	66.1 ± 9.7
Liver	95.7 ± 2.1	98.0 ± 1.7	89.0 ± 4.5	93.0 ± 3.2	74.5 ± 5.7	77.5 ± 7.2	83.0 ± 5.2	88.7 ± 3.8	64.0 ± 6.3	68.8 ± 7.6
Spine	95.4 ± 1.3	96.3 ± 1.3	86.8 ± 3.7	88.3 ± 3.1	69.4 ± 7.4	68.2 ± 6.7	81.1 ± 5.0	82.7 ± 4.7	59.9 ± 8.2	57.8 ± 6.0
TrueBeam STx										
Lung (SABR)	94.5 ± 4.0	97.2 ± 2.8	90.7 ± 5.3	91.8 ± 5.5	73.8 ± 7.4	73.3 ± 8.2	88.2 ± 5.5	89.7 ± 5.5	68.4 ± 7.6	69.1 ± 7.9
Spine (SABR)	97.1 ± 2.1	98.5 ± 0.9	93.4 ± 4.1	95.7 ± 2.0	82.5 ± 7.2	85.6 ± 4.1	90.6 ± 5.0	93.3 ± 2.5	76.4 ± 8.1	80.2 ± 4.2
Liver (SABR)	97.7 ± 2.1	98.6 ± 1.1	93.2 ± 4.8	95.1 ± 3.0	80.8 ± 8.5	83.3 ± 6.7	90.2 ± 5.5	92.5 ± 4.1	74.6 ± 9.5	76.9 ± 8.0
Brain	94.4 ± 2.5	99.1 ± 0.8	89.4 ± 3.2	95.4 ± 2.5	77.3 ± 5.9	80.1 ± 9.5	86.5 ± 3.9	92.8 ± 3.6	71.5 ± 5.9	73.2 ± 10.2
H&N	93.6 ± 1.6	95.9 ± 2.3	86.9 ± 2.7	89.6 ± 5.0	70.0 ± 4.3	73.3 ± 9.2	82.8 ± 3.3	85.2 ± 5.7	61.4 ± 4.7	65.1 ± 9.2

MC: MapCHECK2 measurements; AC: ArcCHECK measurements; H&N: Head and neck; PP: Primary plan; BP: Boost plan; SABR: Stereotactic ablative radiotherapy.

For the VMAT plans with C‐series linac, the MapCHECK2 measurements indicated that the H&N plans showed the lowest gamma passing rates with 3%/3 mm and 2%/2 mm, while the prostate primary plans showed the lowest gamma passing rates with the rest of the gamma criteria. However, the ArcCHECK measurements indicated that the H&N VMAT plans consistently showed the lowest gamma passing rates, regardless of the gamma criteria. Both the MapCHECK2 and the ArcCHECK measurements indicated that the local gamma passing rates of the liver plans were the highest in general. The gamma passing rates with the MapCHECK2 array were generally coincident with those from the ArcCHECK array.

For the TrueBeam STx VMAT plans, both the MapCHECK2 and ArcCHECK measurements indicated that the H&N plans showed the lowest gamma passing rates. Except for gamma passing rates with 3%/3 mm, both the MapCHECK2 and ArcCHECK measurements indicated that the spine SABR VMAT plans showed the highest values for gamma passing rates. Similar to the results with the C‐series linac, the gamma passing rates for the MapCHECK2 array were coincident with those for the ArcCHECK array.

### 3.3 Differences in the mechanical parameters between the original VMAT plans and the log files

The mechanical parameter differences between the original VMAT plans and the log files recorded during the VMAT deliveries are shown in Table 3.

**Table 3:** Differences in mechanical parameters between the original VMAT plans and log files during plan delivery

Treatment site	MLC positioning error (mm)	Gantry angle error (˚)	Monitor unit error (MU)
C‐series linac			
Head and neck	0.19 ± 0.06	0.05 ± 0.00	0.11 ± 0.09
Prostate (primary plan)	0.06 ± 0.15	0.04 ± 0.00	0.06 ± 0.02
Prostate (boost plan)	0.03 ± 0.01	0.04 ± 0.00	0.06 ± 0.01
Liver	0.12 ± 0.10	0.04 ± 0.00	0.07 ± 0.03
Spine	0.07 ± 0.06	0.05 ± 0.01	0.13 ± 0.05
TrueBeam STx			
Lung (SABR)	0.01 ± 0.01	0.01 ± 0.00	0.40 ± 0.03
Spine (SABR)	0.02 ± 0.01	0.03 ± 0.00	0.25 ± 0.09
Liver (SABR)	0.03 ± 0.02	0.04 ± 0.02	0.19 ± 0.11
Brain	0.04 ± 0.02	0.03 ± 0.00	0.05 ± 0.04
Head and neck	0.09 ± 0.01	0.03 ± 0.00	0.02 ± 0.01

SABR: Stereotactic ablative radiotherapy.

For the plan delivery with the C‐series linac, the MLC positioning errors were largest when delivering the H&N VMAT plans, while those differences were the lowest when delivering prostate boost plans. The MU delivery errors were largest for spine VMAT plans, while they were smallest for prostate plans.

For dose delivery with the TrueBeam STx system, the MLC positioning errors were largest for the H&N VMAT plans and were consistent with the C‐series linac results, while those errors were the smallest for the lung SABR VMAT plans. However, the MU delivery errors showed the highest values for the lung SABR VMAT plans and the lowest values for the H&N VMAT plans. The gantry positional error was largest for the liver SABR VMAT plans and was smallest for the lung SABR VMAT plans. In general, the MLC positional errors and the gantry positional errors in the TrueBeam STx system were smaller than those in the C‐series linac. The MU delivery errors in the SABR VMAT plans with the TrueBeam STx system were generally larger than those in the C‐series linac system.

### 3.4 Correlation between the local gamma passing rates and the modulation indices

Spearman's rank correlation coefficients between the modulation index values and the local gamma passing rates acquired with the C‐series linac are shown in Table 4 with their corresponding *P* values. Only *r* values with *P* < 0.05 are shown.

**Table 4:** Spearman's rank correlation coefficients (*r*) between MIs and the local gamma passing rates for the C‐series linac system

	MI_t_	MI_c_	MCS_v_	LTMCS	MI_SPORT_	PA	PI	PM	PMU
*r* (*p*) of MapCHECK2									
3%/3 mm	−0.610 (<0.001)	−0.624 (<0.001)	0.504 (<0.001)	0.623 (<0.001)	−0.622 (<0.001)	−0.269 (0.001)	−0.613 (<0.001)	−0.587 (<0.001)	−0.490 (<0.001)
2%/2 mm	−0.363 (<0.001)	−0.378 (<0.001)	0.444 (<0.001)	0.458 (<0.001)	−0.403 (<0.001)	‐	−0.395 (<0.001)	−0.463 (<0.001)	−0.475 (<0.001)
2%/1 mm	‐	‐	0.353 (<0.001)	0.204 (0.016)	‐	−0.361 (<0.001)	‐	−0.298 (<0.001)	−0.358 (<0.001)
1%/2 mm	−0.236 (0.005)	−0.246 (0.003)	0.265 (0.002)	0.267 (0.001)	−0.230 (0.006)	‐	−0.215 (0.011)	−0.282 (0.001)	−0.310 (<0.001)
1%/1 mm	‐	‐	0.275 (0.001)	‐	‐	−0.323 (<0.001)	‐	−0.231 (0.006)	−0.301 (<0.001)
*r* (*p*) of ArcCHECK									
3%/3 mm	−0.446 (<0.001)	−0.459 (<0.001)	0.527 (<0.001)	0.552 (<0.001)	−0.480 (<0.001)	‐	−0.483 (<0.001)	−0.568 (<0.001)	−0.527 (<0.001)
2%/2 mm	−0.589 (<0.001)	−0.597 (<0.001)	0.457 (<0.001)	0.589 (<0.001)	−0.581 (<0.001)	−0.298 (<0.001)	−0.584 (<0.001)	−0.554 (<0.001)	−0.438 (<0.001)
2%/1 mm	−0.477 (<0.001)	−0.490 (<0.001)	0.538 (<0.001)	0.585 (<0.001)	−0.509 (<0.001)	‐	−0.527 (<0.001)	−0.590 (<0.001)	−0.496 (<0.001)
1%/2 mm	−0.652 (<0.001)	−0.655 (<0.001)	0.343 (<0.001)	0.534 (<0.001)	−0.598 (<0.001)	−0.435 (<0.001)	−0.598 (<0.001)	−0.482 (<0.001)	−0.330 (<0.001)
1%/1 mm	−0.586 (<0.001)	−0.594 (<0.001)	0.444 (<0.001)	0.568 (<0.001)	−0.576 (<0.001)	−0.293 (<0.001)	−0.588 (<0.001)	−0.548 (<0.001)	−0.421 (<0.001)

MI_t_: Modulation index considering mechanical uncertainties in the multi‐leaf collimator (MLC) positions, gantry angle positions, and dose‐rate; MI_c_: Modulation index considering both mechanical uncertainties and dose calculation uncertainty; MCS_v_: Modulation complexity score for volumetric modulated arc therapy (VMAT); LTMCS: Leaf travel modulation complexity score; MI_SPORT_: Modulation index for station parameter optimized radiation therapy; PA: Plan averaged beam area; PI: Plan averaged beam irregularity; PM: Plan averaged beam modulation; PMU: Plan normalized monitor unit.

For the MapCHECK2 measurements, MI_c_ showed the highest correlation to the local gamma passing rate with 3%/3 mm criteria, showing an *r* value of −0.624 (*P* < 0.001). The gamma criterion of 3%/3 mm showed statistically significant correlations to all the modulation indices tested in this study. The 2%/2 mm and 1%/2 mm gamma criteria showed statistically significant correlations to every modulation index in this study, except for PA. For the ArcCHECK measurements, MI_c_ showed the highest correlation to the local gamma passing rate with 1%/2 mm, showing an *r* value of −0.655 (*P* < 0.001). In the case of the ArcCHECK measurements, every gamma criterion tested in this study showed statistically significant correlations to all the modulation indices, except PA. The local gamma passing rates with the ArcCHECK array showed statistically significant correlations to various modulation indices more frequently than did the MapCHECK2 array.

Spearman's rank correlation coefficients between the modulation index values and the local gamma passing rates acquired with the TrueBeam STx system are shown in Table 5, along with their corresponding *P* values. Only *r* values with *P* < 0.05 are shown.

**Table 5:** Spearman's rank correlation coefficients (*r*) between MIs and the local gamma passing rates for the TrueBeam STx system

	MI_t_	MI_c_	MCS_v_	LTMCS	MI_SPORT_	PA	PI	PM	PMU
*r* (*p*) of MapCHECK2									
3%/3 mm	−0.568 (<0.001)	−0.568 (<0.001)	‐	‐	‐	−0.484 (<0.001)	−0.249 (0.012)	‐	‐
2%/2 mm	−0.586 (<0.001)	−0.585 (<0.001)	‐	‐	‐	−0.564 (<0.001)	−0.340 (0.001)	‐	‐
2%/1 mm	−0.534 (<0.001)	−0.532 (<0.001)	‐	‐	‐	−0.360 (<0.001)	−0.202 (0.044)	‐	‐
1%/2 mm	−0.621 (<0.001)	−0.625 (<0.001)	‐	‐	‐	−0.617 (<0.001)	−0.377 (<0.001)	‐	‐
1%/1 mm	−0.602 (<0.001)	−0.601 (<0.001)	‐	‐	‐	−0.479 (<0.001)	−0.331 (0.001)	−0.198 (0.048)	‐
*r* (*p*) of ArcCHECK									
3%/3 mm	−0.389 (<0.001)	−0.392 (<0.001)	0.210 (0.036)	0.239 (0.017)	−0.232 (0.020)	‐	−0.288 (0.004)	−0.331 0.001)	−0.388 (<0.001)
2%/2 mm	−0.489 (<0.001)	−0.487 (<0.001)	‐	‐	‐	‐	−0.233 (0.020)	−0.216 (0.031)	−0.217 (0.030)
2%/1 mm	−0.463 (<0.001)	−0.459 (<0.001)	‐	‐	‐	‐	‐	‐	‐
1%/2 mm	−0.560 (<0.001)	−0.561 (<0.001)	‐	‐	‐	−0.300 (0.002)	−0.323 (0.001)	−0.236 (0.018)	‐
1%/1 mm	−0.559 (<0.001)	−0.555 (<0.001)	‐	‐	‐	−0.218 (0.029)	‐	‐	‐

MI_t_: Modulation index considering mechanical uncertainties in the multi‐leaf collimator (MLC) positions, gantry angle positions, and dose‐rate; MI_c_: Modulation index considering both mechanical uncertainties and dose calculation uncertainty; MCS_v_: Modulation complexity score for volumetric modulated arc therapy (VMAT); LTMCS: Leaf travel modulation complexity score; MI_SPORT_: Modulation index for station parameter optimized radiation therapy; PA: Plan averaged beam area; PI: Plan averaged beam irregularity; PM: Plan averaged beam modulation; PMU: Plan normalized monitor unit.

For the MapCHECK2 array measurements, MI_c_ showed the highest correlation to the local gamma passing rate with 1%/2 mm and showed an *r* value of −0.625 (*P* < 0.001). The MI_t_, MI_c_, PA, and PI showed statistically significant correlations to the local gamma passing rates with every gamma criterion tested in this study. For the ArcCHECK array measurements, MI_c_ also showed the highest correlation to the local gamma passing rate with 1%/2 mm and showed an *r* value of −0.561 (*P* < 0.001). The MI_t_ and MI_c_ showed statistically significant correlations to the local gamma passing rates with every gamma criterion tested in this study. The tendencies of the results with the ArcCHECK array were similar with those with the MapCHECK2 array.

### 3.5 Correlation between the mechanical parameter differences and the modulation indices

Spearman's rank correlation coefficients between the modulation index values and the differences in the MLC leaf positions, gantry angles, and delivered MUs between those described in the original VMAT plans and recorded in the log files during plan delivery are shown in Table 6, along with their corresponding *P* values. Only *r* values with *P* < 0.05 are shown.

**Table 6:** Spearman's rank correlation coefficients (*r*) between modulation indices and mechanical parameter differences recorded in the log files during VMAT plan delivery from those described in the original VMAT plans

	MI_t_	MI_c_	MCS_v_	LTMCS	MI_SPORT_	PA	PI	PM	PMU
*r* (*p*) of C‐series linac									
MLC	0.770 (<0.001)	0.747 (<0.001)	‐	−0.324 (<0.001)	0.617 (<0.001)	0.690 (<0.001)	0.604 (<0.001)	‐	‐
Gantry angle	‐	‐	‐	−0.178 (0.036)	0.228 (0.007)	‐	0.215 (0.011)	0.179 (0.034)	0.233 (0.006)
MU	0.611 (<0.001)	0.639 (<0.001)	−0.756 (<0.001)	−0.723 (<0.001)	0.817 (<0.001)	0.183 (0.030)	0.768 (<0.001)	0.805 (<0.001)	0.844 (<0.001)
*r* (*p*) of TrueBeam STx									
MLC	0.708 (<0.001)	0.712 (<0.001)	−0.426 (<0.001)	−0.615 (<0.001)	0.220 (0.028)	0.642 (<0.001)	0.622 (<0.001)	0.572 (<0.001)	‐
Gantry angle	‐	0.197 (0.049)	−0.288 (0.004)	−0.384 (<0.001)	0.254 (0.011)	0.442 (<0.001)	0.362 (<0.001)	0.243 (0.015)	0.224 (0.025)
MU	0.410 (<0.001)	0.409 (<0.001)	0.364 (<0.001)	−0.516 (<0.001)	0.381 (<0.001)	0.646 (<0.001)	0.516 (<0.001)	0.305 (0.002)	0.295 (0.003)

MI_t_: Modulation index considering mechanical uncertainties in the multi‐leaf collimator (MLC) positions, gantry angle positions, and dose‐rate; MI_c_: Modulation index considering both mechanical uncertainties and dose calculation uncertainty; MCS_v_: Modulation complexity score for volumetric modulated arc therapy (VMAT); LTMCS: Leaf travel modulation complexity score; MI_SPORT_: Modulation index for station parameter optimized radiation therapy; PA: Plan averaged beam area; PI: Plan averaged beam irregularity; PM: Plan averaged beam modulation; PMU: Plan normalized monitor unit; MLC: Multi‐leaf collimator; MU: Monitor unit.

For the C‐series linac system, MI_t_ showed the highest correlation to the MLC positional errors and showed an *r* value of 0.770 (*P* < 0.001) among the modulation indices. The MI_c_ correlation to the MLC positional errors also showed an *r* value higher than 0.7 (*r* = 0.747 with *P* < 0.001). For the gantry angle errors, LTMCS, MI_SPORT_, PI, PM, and PMU showed statistically significant correlations. However, those correlations were weak (*r* < 0.3). Every modulation index showed statistically significant correlations to the MU errors. The PMU showed the strongest correlation to the MU errors and showed an *r* value of 0.844 (*P* < 0.001).

For the TrueBeam system, MI_c_ showed the strongest correlation to the MLC positional errors and showed an *r* value of 0.712 (*P* < 0.001) among the modulation indices, same as the results for the C‐series linac system. For the gantry angle, every modulation index showed statistically significant correlations to the gantry angle positioning error, except for MI_t_. However, those correlations were weak and showed *r* values < 0.45. Every modulation index tested in this study showed statistically significant correlations with the MU delivery errors, and the PA showed the strongest correlation with the MU error (*r* = 0.646 with *P* < 0.001).

### 3.6 Correlation of the modulation indices to the dose‐volumetric parameter differences between the original VMAT plans and the VMAT plans reconstructed with the log files

**Figure 1:**
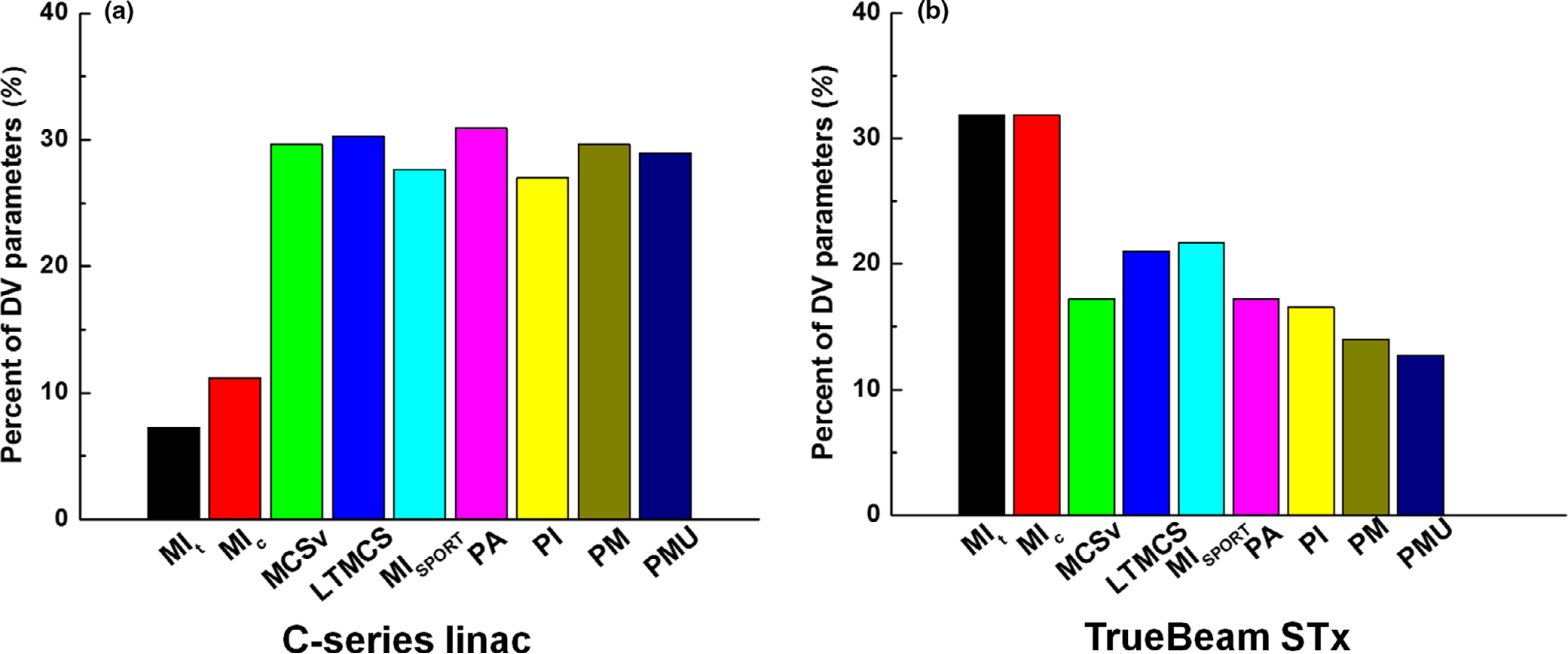
The percent of Spearman's rank correlation coefficients (*r*) of the values of modulation indices to the dose‐volumetric (DV) parameter differences between the original volumetric modulated arc therapy (VMAT) plans and the VMAT plans reconstructed from the log files with *P* < 0.05. The percent values of statistically significant correlation coefficients are shown for VMAT plans generated with the C‐series linac (a) and with the TrueBeam STx (b) systems. The percent values of *r* values of the modulation index values to the differences in the DV parameters between the original VMAT plans and the VMAT plans reconstructed from the log files are shown. A total of nine modulation indices were investigated, which were modulation index considering total mechanical movements (MI_t_), modulation index considering both mechanical and dose calculation uncertainties (MI_c_), modulation complexity score (MCS_v_), leaf travel modulation complexity score (LTMCS), modulation index for station parameter optimized radiation therapy (MI_SPORT_), plan averaged beam area (PA), plan averaged beam irregularity (PI), plan averaged beam modulation (PM), and plan normalized monitor unit (PMU). A total of 152 and 157 DV parameters from VMAT plans with the C‐series linac and the TrueBeam STx systems were investigated, respectively.

For each modulation index, the percent of *r* values with *P* < 0.05 is shown in Fig. 1. A total of 152 and 157 dose‐volumetric parameters from VMAT plans with the C‐series linac and the TrueBeam STx systems were investigated, respectively.

In the case of the C‐series linac, PA showed the highest percent of *r* values with *P* < 0.05 (30.9%), while the MI_t_ index showed the lowest percent of *r* values with *P* < 0.05 (7.2%). However, this tendency was opposite in the case of TrueBeam STx. For TrueBeam STx, MI_t_, and MI_c_ showed the highest percent of *r* values (*P* < 0.05) (31.8%), while the PMU showed the lowest percent of *r* values (*P* < 0.05) (12.7%). The PA with TrueBeam STx showed 17.2% of statistically significant *r* values (*P* < 0.05).

## 4 DISCUSSION

In this study, previously suggested modulation indices for VMAT were comprehensively evaluated with various methods to evaluate VMAT delivery accuracy. These were local gamma evaluations, analysis of the log files recorded during VMAT delivery, and clinically relevant dose‐volumetric parameter analysis with VMAT plans reconstructed from the log files. A total of nine modulation indices were analyzed, which were MI_t_, MI_c_, MCS_v_, LTMCS, MI_SPORT_, PA, PI, PM, and PMU. As previous studies already reported, the MI_t_, MI_c_, MI_SPORT_, PI, PM, and PMU values increased as the degree of modulation in the VMAT plans increased, while MCS_v_ and LTMCS decreased in this study.^5^, ^6^, ^8^, ^19^, ^20^ However, for the PA, the opposite tendency was observed in this study to that of a previous study by Du et al., showing that an PA values increased with modulation degree.^19^ This was caused by the discordance between the average sizes of the beam apertures of the VMAT plans and the modulation degree of VMAT plans because VMAT plans with various target volumes were analyzed in this study. For example, the H&N plans showed higher modulation than the others, while the average beam apertures were larger than the others in order to accommodate their large target volumes. In the case of lung SABR, the modulation degree was low owing to generally round‐shaped target volumes of the lung SABR and relatively large distance between OARs and the target volumes, while the target volume sizes were small. Therefore, the average beam aperture sizes of the highly modulated VMAT plans in this study (H&N VMAT plans) were large, and the average beam aperture sizes of the lowly modulated VMAT plans in this study (lung SABR VMAT plans) were small. This resulted in an increase in the PA values as the modulation degree of VMAT plans increased. If we analyzed the VMAT plans with similar target volume sizes and various modulation degrees, the values of PA would decrease as the modulation degree increases since highly modulated VMAT plans tends to use small beam segments. However, in this study, we analyzed VMAT plans with various treatment sites, resulting in various target volume sizes, and no tendency of PA value decrease was observed as the modulation degree of the VMAT plans increased. The PA does not seem appropriate when evaluating the modulation degree of VMAT plans at different treatment sites.

In the case of the C‐series linac, every modulation index, except for PA (average beam aperture) and PMU (normalized MU by the fractional prescription dose), showed similar tendencies according to the variation of the modulation degree in the VMAT plans. Most modulation indices indicated that the H&N VMAT plans showed the highest modulation, while the prostate boost VMAT plans showed the lowest modulation among all. In the case of the TrueBeam STx system, all the modulation indices, except for PA and PMU, indicated that the H&N VMAT plans showed the highest modulation, similar to the results with C‐series linac. MI_t_ and MI_SPORT_, which are the modulation indices mainly evaluating the mechanical uncertainty during beam delivery, indicated that the spine SABR VMAT plans showed the lowest modulation. The MCS_v_, LTMCS, PA, PI, and PM which are the modulation indices mainly evaluating the average area of the beam apertures, shape irregularity of the aperture, or the frequency of the multiple small segments, indicated that the lung SABR VMAT plans showed the lowest modulation.^5^, ^6^, ^8^, ^19^

The gamma passing rates with the MapCHECK2 array showed similar results to those with the ArcCHECK array, and both showed the lowest gamma passing rates in the H&N VMAT plans in general. To review the correlations of the modulation indices with the local gamma passing rates, MI_c_ showed the strongest correlations with the gamma passing rates for both the MapCHECK2 and ArcCHECK arrays, as well as in the C‐series linac and TrueBeam STx systems. The MI_c_ seems potentially to be an alternative to gamma evaluation. The gamma passing rates with the C‐series linac showed more statistically significant *r* values with the modulation indices than did the gamma passing rates with the TrueBeam STx system. Since the TrueBeam STx delivers VMAT plans more accurately using the integrated control system (*i.e*. supervisor), than did the C‐series linac, the delivery errors of the TrueBeam STx might be smaller than those of the C‐series linac.^24^ This can also be seen in the mechanical errors from the log files. The smaller delivery errors from the TrueBeam STx system resulted in higher gamma passing rates, as shown in Table 3. Therefore, although the modulation degree of VMAT plans with TrueBeam STx changed significantly, the delivery errors were smaller with the TrueBeam STx than those with the C‐series linac. Therefore, it became hard to find correlations between the modulation index and gamma passing rates and then modulation index should be used carefully with the TrueBeam STx system.

To review the mechanical errors during delivery, the average MLC positional errors of the C‐series linac and the TrueBeam STx were <0.2 and 0.1 mm, respectively. On average, gantry angle positioning errors and MU delivery errors in both the C‐series linac and the TrueBeam STx systems were <0.05° and 0.5 MU, respectively. Since previous studies demonstrated that the MLC positioning errors affect VMAT delivery accuracy more significantly than did the others, that is, gantry angle errors and MU delivery errors, the VMAT delivery accuracy of the H&N VMAT seems worse than the others showing the highest MLC errors for H&N VMAT plans with both the Trilogy and TrueBeam STx systems.^4^, ^10^, ^25^ The lower VMAT delivery accuracy of the H&N VMAT plans compared to the others was also identified based on gamma passing rate results. The MLC positioning errors of the TrueBeam STx were always lower than those of the Trilogy system, which indicated more accurate VMAT delivery of the TrueBeam STx system than the Trilogy system. To review correlations between the mechanical errors during VMAT delivery and the modulation indices, MI_t_ showed the highest correlation to the MLC positioning errors of the C‐series linac (*r* = 0.770 with *P* < 0.001). Meanwhile, MI_c_ showed the highest correlation to the MLC positioning errors of TrueBeam STx (*r* = 0.712 with *P* < 0.001), which was consistent with previous studies.^20^ The MI_t_ and MI_c_ indices seem to be used to predict mechanical errors during VMAT delivery at the planning level.

To review the correlations of modulation indices with the differences in the dose‐volumetric parameters between the original VMAT plans and the VMAT plans reconstructed from the log files, the opposite tendency was observed between the result of C‐series linac and that of the TrueBeam STx system. The percent values of statistically significant correlation coefficients for correlations between MI_t_ and MI_c_ with the dose‐volumetric parameter differences were lower than those for other modulation indices in the C‐series linac. However, the opposite tendency was observed for the TrueBeam STx system. Further study may reveal the cause of this opposite tendency, and these studies will be performed in the future.

Unfortunately, we cannot analyze the clinically unacceptable VMAT plans in this study. Every VMAT plan showed gamma passing rates higher than 90% for global gamma passing rates with a gamma criterion of 2%/2 mm (data are not shown), which was the recommended tolerance level for VMAT by Heilemann et al.^10^ Therefore, we cannot determine the tolerance levels for each modulation index evaluated in this study. By utilizing clinically unacceptable VMAT plans, we could recommend tolerance levels for various modulation indices in the future. Furthermore, a multi‐institutional study will be performed in the near future to comprehensively assess the performance of modulation indices in relation to the measures of VMAT delivery accuracy with a gamma criterion of 3%/2 mm recommended by the AAPM TG 218 report.^26^

To comprehensively review the correlations between the previously suggested modulation indices with the conventional verification methods for VMAT delivery, no modulation index always showed the highest correlations with every verification method for VMAT. Because each verification method evaluating VMAT delivery accuracy (gamma evaluation, log file analysis, and so on) has its own limitations, there is no golden‐reference for the pre‐treatment patient‐specific QA at present. Therefore, any modulation indices cannot always show best performance. With overall evaluation, the MI_c_ generally showed the highest correlations with every verification method for VMAT. The MI_c_ showed the highest correlations with the local gamma passing rates acquired with both the MapCHECK2 and the ArcCHECK arrays for both the C‐series linac and the TrueBeam STx system. The MI_c_ also showed the highest correlations with the MLC errors of the TrueBeam STx systems and the most frequent correlations with statistical significance to the clinically relevant dose‐volumetric parameter differences between calculation and delivery with the TrueBeam STx system. Therefore, MI_c_ seems to be the most appropriate indicator for representing the accuracy of VMAT delivery.

## 5 CONCLUSION

In this study, we comprehensively evaluated various types of modulation indices reported in the previous studies by correlation analysis. To review the correlations between modulation indices and the measures of VMAT delivery accuracy comprehensively, MI_c_ showed best capability to predict the accuracy of VMAT plan delivery. The MI_c_ index demonstrated potential to support or to be an alternative to pre‐treatment patient‐specific QA for VMAT in this study. Since the modulation indices, including the MI_c_, can be calculated at the planning level, adopting the modulation indices in the clinic to verify VMAT plans is expected to reduce resources in busy clinical settings.

## CONFLICT OF INTEREST

The authors have no conflict of interest to declare.

## Supporting information


**Table S1.** Differences in dose‐volumetric parameter between the original VMAT plans and the VMAT plansreconstructed with log files.
